# Human carriage of ESBL/pAmpC-producing *Escherichia coli* and *Klebsiella pneumoniae* in relation to the consumption of raw or undercooked vegetables, fruits, and fresh herbs

**DOI:** 10.1128/spectrum.02849-23

**Published:** 2024-01-11

**Authors:** A. P. Meijs, M. Rozwandowicz, P. D. Hengeveld, C. M. Dierikx, S. C. de Greeff, E. van Duijkeren, J. T. van Dissel

**Affiliations:** 1 Centre for Infectious Disease Control, National Institute for Public Health and the Environment, Bilthoven, the Netherlands; 2 Department of Infectious Diseases and Internal Medicine, Leiden University Medical Center, Leiden, the Netherlands; Health Canada, Ottawa, Canada

**Keywords:** ESBL, *Escherichia coli*, *Klebsiella pneumoniae*, fruits, vegetables, herbs, consumption

## Abstract

We investigated to what extent the consumption of raw or undercooked vegetables, fruits, and fresh herbs influences carriage rates of ESBL/pAmpC-producing *Escherichia coli* and *Klebsiella pneumoniae* (ESBL-E/K) in the general population. We assessed long-term carriage and changes in ESBL-E/K prevalence over time, by comparing the results to findings in the same population 5 years earlier. Between July and December 2021, participants sent in two fecal samples and questionnaires, 3 months apart. Food frequency questionnaires were sent on a monthly basis. Fecal samples were cultured and screened for ESBL-E/K, and phenotypically positive isolates were sequenced. Multivariable logistic regression models were established to assess the association between the consumption of fresh produce and ESBL-E/K carriage. The ESBL-E/K prevalence was 7.6% [41/537; 95% confidence interval (CI): 5.7–10.2] in the first sampling round and 7.0% (34/489; 95% CI: 5.0–9.6) in the second. Multivariable models did not result in statistical significance for any of the selected fruit and vegetable types. Trends for increased carriage rates were observed for the consumption of raspberry and blueberry in the summer period. ESBL-E/K prevalence was comparable with the prevalence in the same cohort 5 years earlier (7.5%; 95% CI: 5.6–10.1%). In six persons (1.2%) a genetically highly homologous ESBL-E/K was found. In conclusion, the contribution of the consumption of raw fruits, vegetables, and herbs to ESBL-E/K carriage in humans in the Netherlands is probably low. Despite COVID-19 containment measures (e.g., travel restrictions, social distancing, and hygiene) the ESBL-E/K prevalence was similar to 5 years earlier. Furthermore, indications for long-term carriage were found.

**IMPORTANCE:**

ESBL-producing bacteria are resistant against important classes of antibiotics, including penicillins and cephalosporines, which complicates treatment of infections. Food is one of the main routes of transmission for carriage of these bacteria in the general population. Although fruits, vegetables, and herbs are generally less frequently contaminated with ESBL-producing bacteria compared to meat, exposure might be higher since these products are often eaten raw or undercooked. This research showed that the contribution of the consumption of raw or undercooked fresh produce to ESBL-E/K carriage in the general Dutch population was low. No specific types of fruit or vegetables could be identified that gave a higher risk of carriage. In addition, we demonstrated the presence of genetically highly homologous ESBL-E/K in six persons after a period of 5 years, indicative for long-term carriage.

## INTRODUCTION

Antimicrobial resistance (AMR) is a global threat to the adequate treatment of bacterial infections in both humans and animals. Infections with antibiotic-resistant bacteria, including those caused by ESBL- and plasmid-mediated AmpC-producing Enterobacterales (ESBL-E), are considerably more complex to treat than infections with susceptible microorganisms and result in poorer health outcomes, as the choice of antibiotics is limited (1). In the Netherlands, the prevalence of ESBL-E carriage in the general population is approximately 4–10% (2–4). The primary mode of transmission is via contact with other humans, which was estimated to account for 60% of carriage, whereas food products accounted for 19% of ESBL-E carriage (5). Indeed, ESBL-E are frequently detected in livestock and meat, particularly chicken (6), prompting extensive monitoring of animals and products of animal origin at the national and international levels (7, 8). Although less frequently reported, ESBL-E carriage has also been linked to the consumption of raw vegetables (5). Vegetables and fruits can become contaminated with antimicrobial-resistant bacteria via soil, irrigation water, or by applying organic fertilizer (9). After harvesting, contamination might occur by human handling at the processing, distribution, or preparation stage. Furthermore, antimicrobials are also regularly used as pesticides in plant agriculture, and antimicrobial residues may remain on the food at levels that pose a threat to human health. Several studies from around the world have found contamination with ESBL-E on vegetables, fruits, and herbs (10–16). Also, fresh produce that is sold in the Netherlands has been tested positive for ESBL-E, including bean sprouts, several vegetable types, and imported herbs (17–21). Since 2020 the yearly monitoring of ESBL-producing *Escherichia coli* in meat in the Netherlands has been expanded with vegetables and mushrooms (7). In the first 2 years, prevalence rates in vegetables were 0.2% and 0.3%, respectively.

Food of plant origin appears to be less frequently colonized with antimicrobial-resistant bacteria compared to meat products. However, their role as a reservoir might be important, particularly as many of these products are ready-to-eat and thus consumed without any preparation, such as washing or heating. Previous research revealed that people on a vegetarian diet did not have a lower risk of ESBL-producing *E. coli* or *Klebsiella pneumoniae* (ESBL-E/K) carriage compared to persons who consumed meat at least three times per week (22). The present cohort study followed up on these persons and assessed to what extent the consumption of raw or undercooked vegetables, fruits, and fresh herbs influenced carriage rates of ESBL-E/K. Second, we assessed long-term carriage as well as whether the overall ESBL-E/K prevalence differed during the COVID-19 pandemic, by comparing it to earlier findings in the same population.

## MATERIALS AND METHODS

Participants were recruited from a previous cross-sectional study performed in the Netherlands in 2015–2017 (22). In this population-based study, vegetarians were oversampled in order to compare ESBL-E/K prevalence between persons with different diets. In June and July 2021, 1,541 participants were contacted again via email and received information and an invitation to participate in the present study. The Medical Research Ethics Committee Utrecht reviewed this study and granted it an official exemption for approval under the medical research involving human subjects act (WMO) (number 21-302/C). All participants were aged 18 years or older and provided informed consent via a digital form.

Participants were requested to provide two fecal samples: one at baseline (July–September 2021) and one at the end of the study period 3 months later (October–December 2021). Samples were collected at home by the participants themselves, supported by a detailed instruction leaflet (see supplementary material). Samples were stored at room temperature until they were sent to our laboratory via regular mail, preferably on the same day. The baseline measurement included a detailed web-based questionnaire asking about general characteristics, grocery shopping, dietary habits, hygiene, health, hospitalization, medication use, travel, leisure activities, occupation, and contact with animals. During the study, participants received four food frequency questionnaires (FFQs) on a monthly basis, focusing on the frequency of consumption of specific raw or undercooked fruits, vegetables, and fresh herbs in the past 7 days. Together with the second fecal sample a questionnaire regarding the participant’s health, medication use, travel, leisure activities, contacts, and hygiene in the preceding 3 months was administered in order to identify whether risks for ESBL-E/K were encountered during the three-month study period.

### Microbiology and whole-genome sequencing

On the day of arrival at the laboratory, the fecal samples were stored at 4°C until further processing, which took place within 2 days of sample collection in 81.0% (*n* = 831/1,026) of the samples. Two samples were processed more than 1 week after the sample was taken, both after 9 days, and for 68 samples (6.6%), the date of collection was unknown.

A similar culture protocol for the detection of ESBL-E/K was used as in the cross-sectional study (22). The fecal samples were plated directly on Brilliance *E. coli*/coliform Selective Agar (Oxoid) with 1 mg/L cefotaxime (Sigma) and without cefotaxime (BECSA^+^ and BECSA^−^). A sterile cotton swab stirred through the fecal sample was placed into a sterile tube with 2 mL of Luria Bertani broth (MP Biomedicals) supplemented with 1 mg/L cefotaxime for selective enrichment. The broth and plates were incubated overnight at 37°C. The selective enrichment with cefotaxime was plated on BECSA^+^ for all samples, and incubated overnight at 37°C. If plates with cefotaxime showed suspected growth of ESBL-E/K, three colonies per sample (preferably from the plate without selective enrichment) were plated on Columbia Agar with 5% sheep blood (Oxoid) for further testing. The species of pink-colored colonies were confirmed using Matrix Assisted Laser Desorption/Ionization Time-Of-Flight Mass Spectrometry (MALDI-TOF MS) (Bruker).

The phenotypically positive ESBL-E/K isolates were sequenced using Illumina short-read sequencing. DNA isolations were performed using Maxwell RSC Cultured Cells DNA kit (Promega). Sequencing was performed in-house on a NextSeq platform (Illumina). Sequences were assembled using the Juno-Assembly pipeline (https://github.com/RIVM-bioinformatics/juno-assembly). Presence of resistance genes and plasmid types was determined with ABRicate (https://github.com/tseemann/abricate) using ResFinder and PlasmidFinder databases, respectively (23, 24). *E. coli* and *K. pneumoniae* Multi Locus Sequence Type (MLST) was determined with MLST software (https://github.com/tseemann/mlst). If a participant carried the same ESBL/pAmpC gene and bacterial ST in both samples or the same gene and ST were also found in the cross-sectional study in 2015–2017, a selection of two isolates was sequenced with long-read sequencing (Oxford Nanopore), in order to determine the plasmid type. DNA isolations for long-read sequencing were performed using the DNeasy Blood and Tissue kit (Qiagen), supplemented with an RNA-depletion step, using RNase A (Thermo Scientific) according to the manufacturer’s protocol. Library preparation was done using the SQK-RBK004 rapid barcoding kit (Oxford Nanopore). The labeled DNA was loaded on an R9.4.1 flow cell and run on the GridIon using the Super Accuracy base calling protocol. Isolates for which both short- and long-read sequences were available were hybrid assembled using FullForce Plasmid Assembler (https://github.com/NorwegianVeterinaryInstitute/FullForcePlasmidAssembler).

### Data analyses

Descriptive statistics were used to summarize the general characteristics of the study population. ESBL-E/K prevalence, including Wilson’s 95% confidence intervals (CIs), was determined for the two sample moments separately and a cumulative prevalence was calculated using the results of the two samples combined for persons that provided both. The weekly consumption of fresh produce was calculated by the number of days in the past week that the product was eaten, multiplied by the average number of serving spoons, pieces, or number of hands (depending on the product) per day, for every FFQ. The average weekly consumption of every food item was calculated by taking the mean of the FFQs provided, per participant. A selection of soft fruits (strawberries, blackberries, raspberries, other berries, and cherries) that are produced in the Netherlands in the summer months (July–September) but are mainly imported from abroad in the other seasons, was analyzed separately for the summer and for the other seasons.

The correlation between the consumption of fruits, vegetables, and herbs was assessed in a correlation matrix using Spearman correlation coefficients. The logistic regression models were established with the cumulative ESBL-E/K carriage, as the outcome of interest. Only data of participants who supplied both fecal samples were included in the models. The dependent variables for the consumption of vegetables, fruits, and herbs were dichotomized as consumption (average consumption of >0 serving spoons, pieces, or hands per week) versus no consumption (average consumption of 0 serving spoons, pieces, or hands per week). Univariable analyses were performed for all food items of interest and for known risk factors for ESBL-E/K carriage being hospitalization, antibiotic use and travel, as well as potential risk factors being diet (vegetarian, non-vegetarian, and pescatarian) and kitchen hygiene. Food items were further analyzed in multivariable logistic regression models, while adjusting for risk factors with a *P* value < 0.20. In case food items showed moderate or strong correlation (Spearman correlation coefficient ≥ 0.40), interaction terms were added to the models (25). Models were built for fruits, and vegetables/herbs separately. A *P* value < 0.05 was used to determine significance. A Bonferroni adjustment for multiple testing was applied. Analyses were performed using SAS version 9.4 (SAS Institute Inc., Cary, NC, USA) and R version 4.2.0 (R Foundation for Statistical Computing, Vienna, Austria).

## RESULTS

Following the invitation and two reminders, 583 persons signed informed consent, resulting in a response rate of 37.8% (*n* = 583/1,541). The first fecal sample and general questionnaire were returned by 537 participants between July and September 2021, 489 participants provided the second sample between October and December 2021. In total, 85.5% of the participants (*n* = 459) completed all four FFQs, 9.5% completed three, 3.0% filled out two, and 2.1% only one. The median age of participants was 56 years (min–max: 24–88) and 75% were female. Most participants (57.7%; *n* = 310) were vegetarians (including vegans), 125 (23.3%) were pescatarians (vegetarians who eat fish) and 102 (19.0%) ate both meat and fish (non-vegetarians). More characteristics can be found in Table 1 and in Table S1 and S2. The ESBL-E/K prevalence based on the first fecal sample was 7.6% (41/537; 95% CI: 5.7–10.2) and 7.0% based on the second sample (34/489; 95% CI: 5.0–9.6). The difference in ESBL-E/K prevalence between the two sample moments was non-significant (*P* value 0.7). In total, 10.8% (53/489; 95% CI: 8.4–13.9) of participants who provided both fecal samples were found ESBL-E/K positive at one or both time points. Four persons carried an ESBL-producing *K. pneumoniae* (0.8%; 95% CI: 0.3–2.1), all others carried *E. coli*.

**TABLE 1:** Logistic regression analysis of the average weekly consumption^*a*^ of raw or undercooked vegetables, fruits, and fresh herbs and ESBL-E/K carriage

			ESBL-E/K		Logistic regression^*b*^						
		All (*n* = 489)	Negative (*n* = 436)	Positive (*n* = 53)	Univariable			Multivariable^*c*^		
	Cumulative prevalence^*d*^	*n* (%)	*n* (%)	*n* (%)	OR	95% CI	*P* value	OR	95% CI	*P* value
Total	10.8%									
General risk factors										
Hospitalization^*e*^	11.5%	26 (5.3)	23 (5.3)	3 (5.7)	1.09	0.32–3.76	0.893			
Antibiotic use in last 3 months^*f*^	13.8%	58 (11.9)	50 (11.5)	8 (15.1)	1.39	0.62–3.11	0.429			
Diet										
Vegetarian	11.0%	282 (57.7)	251 (57.6)	31 (58.5)	1.13	0.51–2.46	0.768			
Non-vegetarian	9.9%	91 (18.6)	82 (18.8)	9 (17.0)	Ref.					
Pescatarian	11.2%	116 (23.7)	103 (23.6)	13 (24.5)	1.15	0.47–2.82	0.760			
Meat consumption per week										
Never	11.1%	398 (81.4)	354 (81.2)	44 (83.0)	0.94	0.40–2.20	0.89			
<3 times	6.5%	31 (6.3)	29 (6.7)	2 (3.8)	0.52	0.10–2.68	0.44			
≥3 times	11.7%	60 (12.3)	53 (12.2)	7 (13.2)	Ref.					
How often do you buy organic fruits and vegetables										
I do not know	12.5%	7 (1.4)	6 (1.4)	1 (1.9)						
Always/usually	11.7%	72 (14.7)	59 (13.5)	13 (24.5)	*2.04*	*0.92–4.56*	*0.081*	1.67	0.72–3.88	0.230
Regularly/sometimes	7.2%	256 (52.4)	232 (53.2)	24 (45.3)	0.96	0.49–1.89	0.903	0.90	0.45–1.83	0.776
Rarely/never	6.4%	154 (31.5)	139 (31.9)	15 (28.3)	Ref.			Ref.		
Hand washing frequency before food preparation										
Always/usually	12.0%	291 (59.5)	256 (58.7)	35 (66.0)	Ref.			Ref.		
Regularly/sometimes	7.1%	168 (34.4)	156 (35.8)	12 (22.6)	*0.56*	*0.28–1.12*	*0.100*	*0.50*	*0.24–1.02*	*0.058*
Rarely/never	20.0%	30 (6.1)	24 (5.5)	6 (11.3)	1.83	0.70–4.78	0.219	1.51	0.52–4.43	0.453
Travel^*g*^										
UNK	16.7%	6 (1.2)	5 (1.2)	1 (1.9)						
No travel, travel to Western/Northern Europe, North America, Australia, or New Zeeland	10.6%	404 (82.6)	361 (82.8)	43 (81.1)	Ref.			Ref.		
Travel to Southern/Eastern Europe	9.0%	67 (13.7)	61 (14.0)	6 (11.3)	0.83	0.34–2.02	0.676	0.75	0.30–1.89	0.544
Travel to Africa, Asia, or Latin America	25.0%	12 (2.5)	9 (2.1)	3 (5.7)	*2.80*	*0.73–10.74*	*0.133*	*2.71*	*0.64–11.42*	*0.175*
Fruit										
Strawberry	11.3%	353 (72.2)	313 (71.8)	40 (75.5)	1.21	0.63–2.34	0.573			
Apple	11.5%	392 (80.2)	347 (79.6)	45 (84.9)	1.44	0.66–3.17	0.362			
Pear	10.0%	221 (45.2)	199 (45.6)	22 (41.5)	0.85	0.47–1.51	0.568			
Grape	10.5%	323 (66.1)	289 (66.3)	34 (64.2)	0.91	0.50–1.65	0.757			
Cherry	12.9%	202 (41.3)	176 (40.4)	26 (49.1)	1.42	0.80–2.52	0.227			
Raspberry	14.3%	230 (47.0)	197 (45.2)	33 (62.3)	**2.00**	**1.11–3.60**	**0.020**	*1.88*	*0.98–3.59*	*0.057*
Blackberry	13.3%	173 (35.4)	150 (34.4)	23 (43.4)	*1.46*	*0.82–2.61*	*0.198*	1.10	0.57–2.11	0.773
Blueberry	12.5%	329 (67.3)	288 (66.1)	41 (77.4)	*1.76*	*0.90–3.44*	*0.101*	1.23	0.59–2.58	0.581
Other berry	14.8%	108 (22.1)	92 (21.1)	16 (30.2)	*1.62*	*0.86–3.04*	*0.135*	1.29	0.64–2.59	0.478
Mandarin	11.5%	295 (60.3)	261 (59.9)	34 (64.2)	1.19	0.66–2.16	0.560			
Orange	11.8%	203 (41.5)	179 (41.1)	24 (45.3)	1.18	0.67–2.10	0.565			
Apricot	11.6%	86 (17.6)	76 (17.4)	10 (18.9)	1.10	0.53–2.29	0.795			
Peach	11.8%	170 (34.8)	150 (34.4)	20 (37.7)	1.16	0.64–2.08	0.631			
Nectarine	10.2%	166 (34.0)	149 (34.2)	17 (32.1)	0.91	0.49–1.67	0.761			
Plum	12.4%	202 (41.3)	177 (40.6)	25 (47.2)	1.31	0.74–2.32	0.360			
Dried fruit	11.6%	353 (72.2)	312 (71.6)	41 (77.4)	1.36	0.69–2.67	0.375			
Vegetables and herbs										
Cabbage	10.2%	266 (54.4)	239 (54.8)	27 (50.9)	0.86	0.48–1.52	0.593			
Iceberg lettuce	12.1%	264 (54.0)	232(53.2)	32 (60.4)	1.34	0.75–2.40	0.324			
Arugula	11.4%	272 (55.6)	241 (55.3)	31 (58.5)	1.13	0.64–2.02	0.669			
Packed lettuce	10.7%	364 (74.4)	325 (74.5)	39 (73.6)	0.95	0.50–1.82	0.880			
Ready-to-eat salad	12.6%	151 (30.9)	132 (30.3)	19 (35.9)	1.29	0.71–2.34	0.408
Coleslaw	8.8%	148 (30.3)	135 (31.0)	13 (24.5)	0.73	0.38–1.40	0.338
Tomato	11.0%	465 (95.1)	414 (95.0)	51 (96.2)	1.36	0.31–5.93	0.687
Cucumber	11.2%	439 (89.8)	390 (89.5)	49 (92.5)	1.45	0.50–4.19	0.498
Zucchini	10.8%	213 (43.6)	190 (43.6)	23 (43.4)	0.99	0.56–1.76	0.969
Bell pepper	11.9%	386 (78.9)	340 (78.0)	46 (86.8)	*1.86*	*0.81–4.24*	*0.143*	1.35	0.56–3.25	0.507
Carrot	11.2%	376 (76.9)	334 (76.6)	42 (79.3)	1.17	0.58–2.35	0.667			
Celery	14.1%	128 (26.2)	110 (25.2)	18 (34.0)	*1.52*	*0.83–2.79*	*0.178*	1.27	0.66–2.46	0.475
Endive	9.4%	128 (26.2)	116 (26.6)	12 (22.6)	0.81	0.41–1.59	0.536			
Spinach	10.8%	222 (45.4)	198 (45.4)	24 (45.3)	1.00	0.56–1.76	0.986			
Kale	8.0%	50 (10.2)	46 (10.6)	4 (7.6)	0.69	0.24–2.01	0.498			
Chicory	8.6%	162 (33.1)	148 (33.9)	14 (26.4)	0.70	0.37–1.33	0.274			
Radish	11.5%	165 (33.7)	146 (33.5)	19 (35.9)	1.11	0.61–2.01	0.731			
Spring onions	11.3%	318 (65.0)	282 (64.7)	36 (67.9)	1.16	0.63–2.13	0.640			
Bean sprouts	9.5%	137 (28.0)	124 (28.4)	13 (24.5)	0.82	0.42–1.58	0.550
Alfalfa	15.1%	53 (10.8)	45 (10.3)	8 (15.1)	1.55	0.69–3.48	0.294
Other sprout vegetables	13.4%	67 (13.7)	58 (13.3)	9 (17.0)	1.33	0.62–2.88	0.463			
Herb plants	11.2%	322 (65.9)	286 (65.6)	36 (67.9)	1.11	0.60–2.04	0.736			
Boxed fresh herbs	12.6%	222 (45.4)	194 (44.5)	28 (52.8)	1.40	0.79–2.47	0.251			

^*a*^ The average consumption was dichotomized as >0 serving spoons, pieces or hands per week versus 0 serving spoons, pieces, or hands per week.

^*b*^ Bold text indicates a *P* value < 0.05. Italic text indicates a *P* value < 0.20.

^*c*^ Adjusted for buying organic fruits and vegetables, hand washing frequency before food preparation and travel.

^*d*^ Based on two sample moments, 3 months apart.

^*e*^ Hospitalized in Dutch hospital in 6 months before first fecal sample or between first and second samples.

^*f*^ Antibiotic use in 3 months before first fecal sample or between first and second samples.

^*g*^ Travel in 6 months before first fecal sample or between first and second samples.

**Fig 1:**
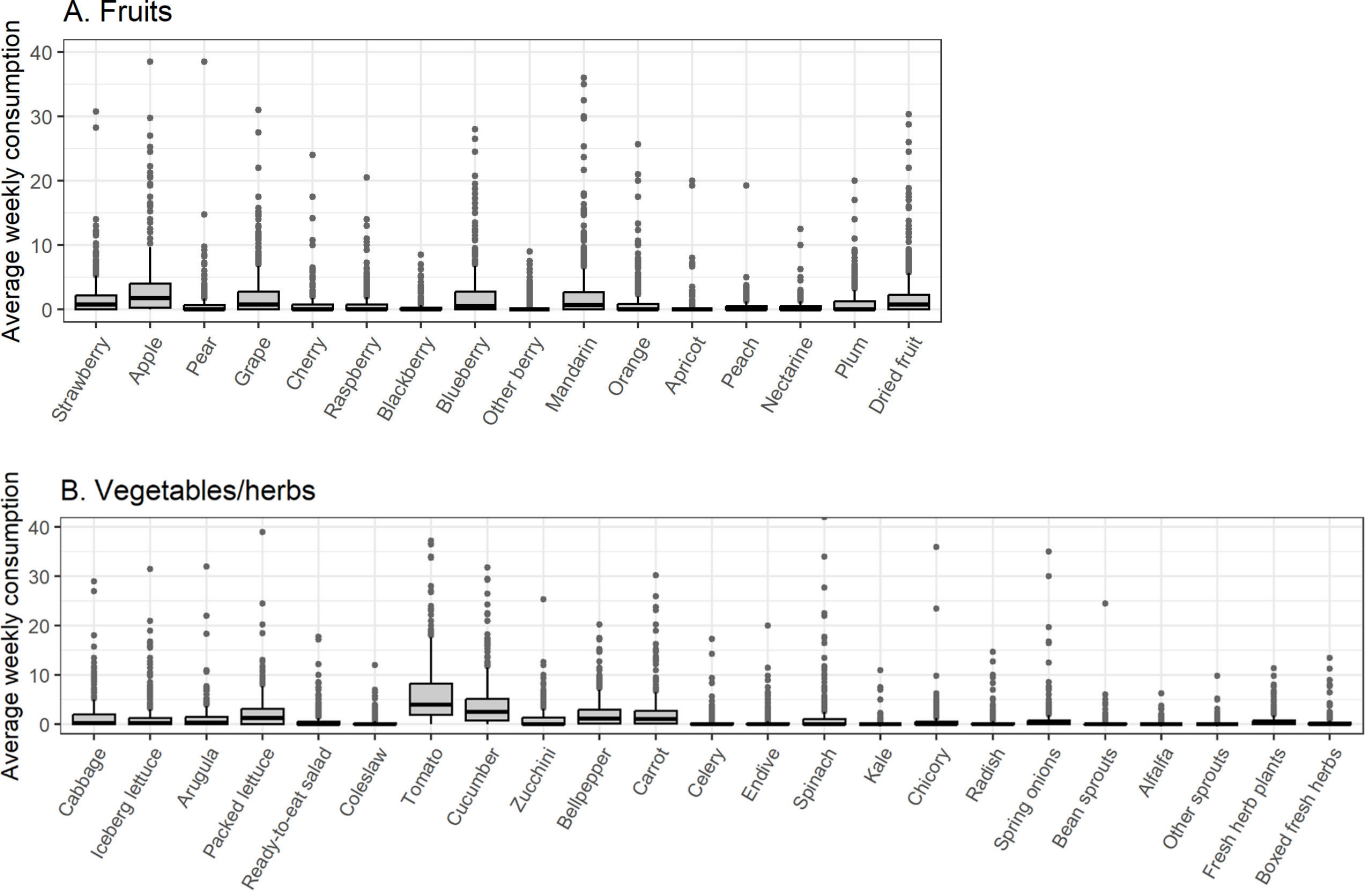
Box plots for the average weekly consumption of raw or undercooked (**A**) fruits, and (**B**) vegetables and fresh herbs (*n* = 489). The average weekly consumption of every food item was calculated per participant, by taking the mean consumption from all food frequency questionnaires. Four outliers are removed [iceberg lettuce (1), spinach (1), orange (1), and apple (1)] with average weekly consumption >40.

The average weekly consumption of 23 types of vegetables and fresh herbs and 16 types of fruit for the 489 participants who supplied both fecal samples is shown in Fig. 1; Fig. S1 and S2. The frequency distribution was positively skewed and included outliers toward the higher values for the consumption of all food items. Based on the results of the univariable logistic regression analyses bell pepper, celery, raspberry, blackberry, blueberry, and other berries were included in the multivariable models, all with *P* value < 0.20 (see Table 1). No interaction was observed between vegetables or fruits that were selected for multivariable analysis (see Fig. S3 and S4). The multivariable models did not result in statistical significance for any of the selected fruit and vegetable types.

Consumption of soft fruits (strawberries, raspberries, blackberries, blueberries, and other berries) outside of the summer period, when these products are mainly imported from abroad, was not associated with an increased odds of ESBL-E/K carriage, see Table 2. In the summer period, trends for increased carriage rates were observed for the consumption of raspberries and blueberries based on univariable analysis. The consumption of raspberries in the summer period resulted in an increased odds of ESBL-E/K carriage [odds ratio (OR): 2.14; 95% CI: 1.12–4.11; *P* value = 0.022] in multivariable analysis, but not when using the Bonferroni *P* value for significance (*P* value < 0.0013).

**TABLE 2:** Logistic regression analysis of the average weekly consumption^*a*^ of soft fruits and ESBL-E/K carriage, separately for the summer and the other seasons

		All (*n* = 489)	ESBL-E/K		Logistic regression^*b*^					
			Negative (*n* = 436)	Positive (*n* = 53)	Univariable			Multivariable^*c*^		
	Cumulative prevalence^*d*^	*N* (%)	*N* (%)	*N* (%)	OR	95% CI	*P* value	OR	95% CI	*P* value
Consumption in summer										
Strawberry	11.2%	331 (67.7)	294 (67.4)	37 (69.8)	1.12	0.60–2.08	0.727			
Cherry	12.6%	198 (40.5)	173 (39.7)	25 (47.2)	1.36	0.77–2.41	0.295			
Raspberry	15.7%	204 (41.7)	172 (39.5)	32 (60.4)	**2.34**	**1.31–4.19**	**0.004**	**2.14**	**1.12–4.11**	**0.022**
Blackberry	14.3%	161 (32.9)	138 (31.7)	23 (43.4)	*1.66*	*0.93–2.96*	*0.088*	1.31	0.70–2.47	0.403
Blueberry	13.3%	301 (61.6)	261 (59.9)	40 (75.5)	**2.06**	**1.07–3.97**	**0.030**	1.39	0.67–2.85	0.375
Other berry	13.5%	96 (19.6)	83 (19.0)	13 (24.5)	1.38	0.71–2.70	0.343			
Consumption in other seasons										
Strawberry	12.7%	142 (29.0)	124 (28.4)	18 (34.0)	1.32	0.72–2.43	0.366			
Cherry	10.0%	10 (2.0)	9 (2.1)	1 (1.9)	0.93	0.12–7.46	0.942			
Raspberry	11.9%	109 (22.3)	96 (22.0)	13 (24.5)	1.17	0.60–2.29	0.638			
Blackberry	11.1%	54 (11.0)	48 (11.0)	6 (11.3)	1.05	0.43–2.59	0.917
Blueberry	9.4%	191 (39.1)	173 (39.7)	18 (34.0)	0.80	0.44–1.46	0.465
Other berry	16.1%	31 (6.3)	26 (6.0)	5 (9.4)	1.67	0.61–4.55	0.317

^*a*^ The average consumption was dichotomized as > 0 hands per week versus 0 hands per week.

^*b*^ Bold text indicates a p-value <0.05. Italic text indicates p-value <0.20.

^*c*^ Adjusted for buying organic fruits and vegetables, hand washing frequency before food preparation and travel.

^*d*^ Based on two sample moments, 3 months apart.

*bla*_CTX-M-15_ was the predominant ESBL/pAmpC gene at both sampling moments, found in 41.5% (*n* = 17/41) of ESBL-E/K positive participants in the first fecal sample and 32.4% (*n* = 11/34) in the second sample, followed by *bla*_DHA-1_ in 14.6% (*n* = 6/41) and 23.5% (*n* = 8/34), respectively (see Table 3; Table S3). In all four persons carrying an ESBL-producing *K. pneumoniae* an SHV-gene was found. The most frequently found *E. coli* STs were 69 (*n* = 15), 10 (*n* = 13), and 131 (*n* = 10). Of the 34 persons that were ESBL-E/K positive at the second sampling moment, 15 (44.1%) carried the same ESBL/pAmpC gene in the same *E. coli* type compared to 3 months earlier. Thirteen participants (2.7%) became ESBL-E/K carriers during the study period and six remained positive but carried a different ESBL gene in a different bacterial strain (*n* = 5) or the same gene in a different *E. coli* ST (*n* = 1). A description of general characteristics and known risk factors of these persons is shown in Table S4. Three of these 19 persons were hospitalized in the period between the two samples, one of whom also received antibiotics. One person traveled outside Europe.

**TABLE 3:** Bacterial sequence types (*E. coli/K. pneumoniae*) ESBL/pAmpC genes and gene locations in participants carrying ESBL-E/K at sample moment 1 and/or sample moment 2, including results of the cross-sectional study (2015–2017)^*a*^

	2015–2017^*b*^^,^^*c*^^,^^*d*^			2021—Sample 1^*b*^^,^^*c*^^,^^*d*^			2021—Sample 2^*b*^^,^^*c*^^,^^*d*^		
Participant	ST^*e*^	ESBL/pAmpC gene	Gene location	ST^*e*^	ESBL/pAmpC gene	Gene location	ST^*e*^	ESBL/pAmpC gene	Gene location
1	-			k17	DHA-1 and SHV-187		-		
2	-			1485	CMY-2		349	DHA-1	
**3**	**131**	**CTX-M-15**	**Chromosome**	**131**	**CTX-M-15**	**Chromosome**	**131**	**CTX-M-15**	
**4**	**69**	**CTX-M-55**	**IncB/O/K/Z**	**69**	**CTX-M-55**	**IncB/O/K/Z**	**69**	**CTX-M-55**	
5	-			69	CTX-M-15		-		
**6**	**-**			**10**	**CTX-M-14**	**Chromosome**	**10**	**CTX-M-14**	**Chromosome**
7	-			-			UNK	CTX-M-55	
**8**	**69**	**CTX-M-15**	**IncF**	**69**	**CTX-M-15**		**69**	**CTX-M-15**	**IncF**
9	-			-			1193	CTX-M-55	
10	-			-			69	CTX-M-15	
11	-			kUNK	SHV-27		-		
12	-			227	CTX-M-15		-		
**13**	**-**			**69**	**CTX-M-27**	**IncF**	**69**	**CTX-M-27**	**IncF**
14	-			349	DHA-1		-		
**15**	**131**	**CTX-M-15**	**Non-typeable plasmid**	**131**	**CTX-M-15**	**Non-typeable plasmid**	**131**	**CTX-M-15**	
16	-			-			38	DHA-1	
**17**	**-**			**38**	**CTX-M-14**	**Chromosome**	**38**	**CTX-M-14**	**Chromosome**
18	-			4981	CTX-M-15		-		
19	-			-			58	CTX-M-55	
20	227	CTX-M-1	IncI1	69	DHA-1		-		
**21**	**-**			**10**	**CTX-M-15, SHV-12**	**Chromosome**	**10**	**CTX-M-15**	**Chromosome**
22	-			14	SHV-12		UNK	CTX-M-15	
**23**	**-**			**131**	**CTX-M-27**	**IncF**	**131, UNK**	**CTX-M-27**	**IncF**
24	-			-			73	TEM-15	
*25*	UNK	*CTX-M-15*		10	*CTX-M-15*		-		
26	UNK	CTX-M-15		1429, 1434, 4774	CTX-M-1, CTX-M-55		-		
27	-			1193	CTX-M-27		k15	SHV-106	
28	-			636	CTX-M-15		-		
29	-			-			156	DHA-1	
30	-			1429	CTX-M-1, CTX-M-55		-		
**31**	**10**	**CMY-2 like**	**IncB/O/K/Z**	**10**	**CMY-2 like**	**IncB/O/K/Z**	**10**	**CMY-2 like**	
32	-			1727	CTX-M-15		-		
**33**	**-**			**10**	**CTX-M-15**	**IncFII**	**10, 409**	**CTX-M-15**	**IncFII**
34	-			69	CTX-M-15		-		
**35**	**-**			**131**	**CMY-2**	**IncI1**	**131**	**CMY-2**	**IncI1**
**36**	**131**	**CTX-M-15**	**IncB/O/K/Z**	**131**	**CTX-M-15**		**131**	**CTX-M-15**	**IncB/O/K/Z**
37	-			1429	CTX-M-55		-		
38	-			10	CTX-M-15		No sample		
39	-			-			23	CTX-M-27	
40	-			10	SHV-12		-		
**41**	**-**			**69, 5662**	**SHV-12**	**IncX3**	**69**	**SHV-12**	**IncX3**
**42**	**-**			**349**	**DHA-1**	**Non-typeable plasmid**	**106, 349**	**CTX-M-65, DHA-1**	**Non-typeable plasmid**
43	-			-			38, 382, 504	CTX-M-15	
44	-			3672	CTX-M-2		-		
45	-			-			38	DHA-1	
46	-			-			349	DHA-1	
47	-			2308	SHV-12		-		
48	-			-			38	CTX-M-15	
49	-			58, k1873	CTX-M-8, CTX-M-15 and SHV-32		69	CTX-M-1	
50	-			10	DHA-1		-		
51	-			69	CTX-M-15		-		
52	-			69	*DHA-1*		10	*DHA-1*	
53	-			-			517	CTX-M-15	
54	-			206, 2325	CTX-M-15		708, 1155	DHA-1	

^*a*^ pAmpC, plasmid mediated AmpC; ST, sequence type; UNK, unknown/non-typeable sequence type.

^*b*^ Three isolates were tested per sample, in some participants more than one sequence type or ESBL/pAmpC gene was found. If multiple genes were found in the same isolate this is indicated with 'and'.

^*c*^ Italic text indicates that the same ESBL/pAmpC gene but different bacterial ST was found at multiple time points. The ESBL/pAmpC gene and bacterial ST were determined with illumina sequencing (2021 samples 1 and 2) or PCR (cross sectional study 2015–2017).

^*d*^ Bold text indicates that the same ESBL/pAmpC gene, bacterial ST and plasmid combination was found at multiple time points. If a participant carried the same ESBL/pAmpC gene and bacterial ST in both samples or the same gene and ST was also found in the cross-sectional study in 2015–2017, a selection of two isolates were long-read sequenced in order to determine the plasmid type.

^*e*^ The *K. pneumoniae* sequence types are indicated with a “k” and underlined; all other sequence types are *E. coli*.

**Fig 2:**
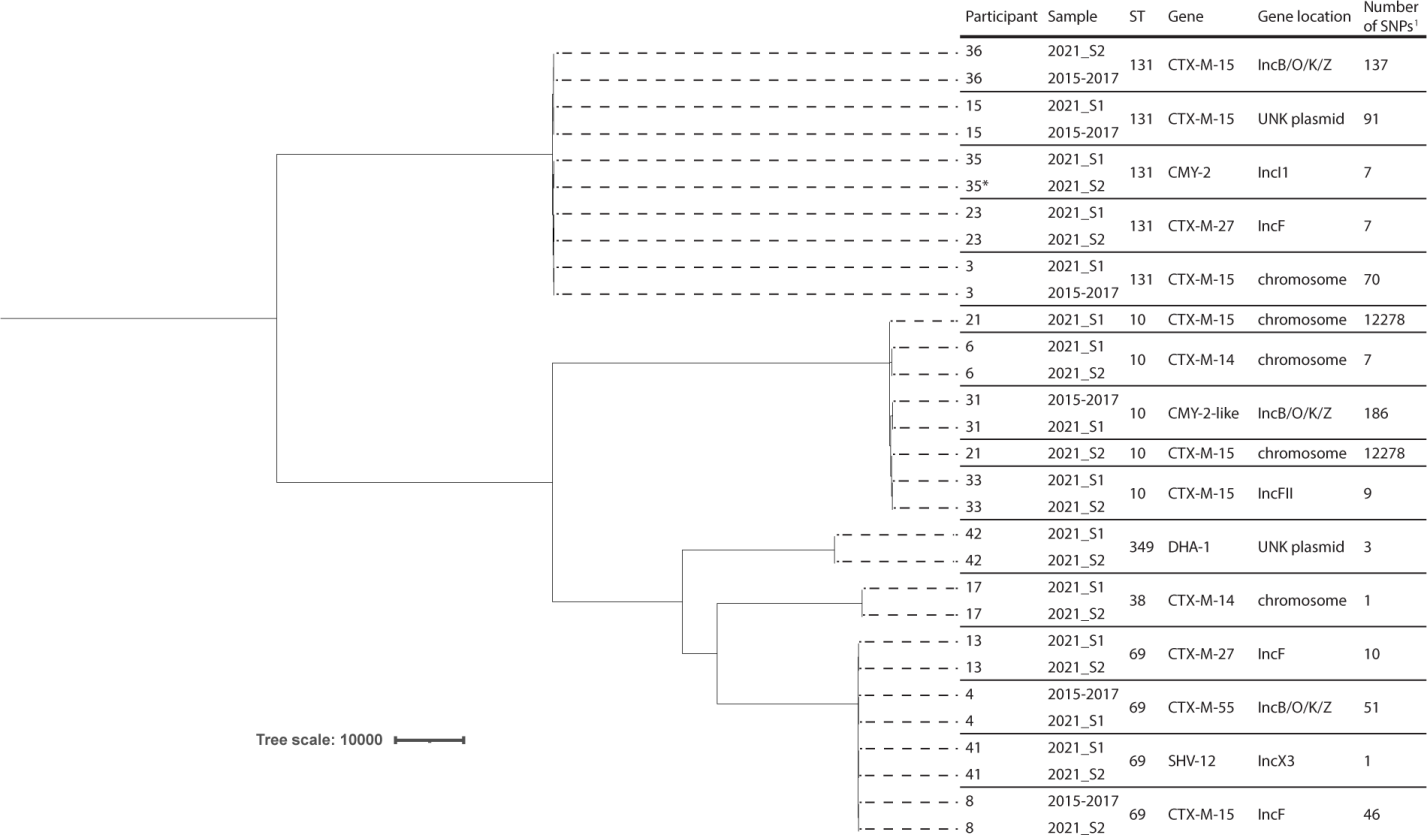
Phylogenetic tree of the *E. coli* core genome of participants who carried the same ESBL/pAmpC and *E. coli* ST in both samples (2021_S1 and 2021_S2, 3 months apart) or who carried the same ESBL/pAmpC and *E. coli* ST in the cross-sectional study in 2015–2017. The location of the ESBL/pAmpC gene was determined after long-read sequencing. Plasmids were identified using PlasmidFinder. The sequences were analyzed using Parsnp (https://github.com/marbl/parsnp), corrected for the presence of repeat regions using Gubbins (https://www.sanger.ac.uk/tool/gubbins/). The phylogenetic tree was visualized with Figtree (http://tree.bio.ed.ac.uk/software/figtree). *Sample 2021_S2 from participant 35 was used as reference. ^1^Number of SNPs difference between two samples of the same participant.

ESBL-E/K prevalence in the previous cross-sectional study performed in 2015–2017 and limited to the cohort included in the present study, was 7.5% (40/532; 95% CI: 5.6–10.1%). Identifying information of five persons could not be matched, these were all ESBL-E/K negative in the present study. Six persons (1.2%) carried the same ESBL/pAmpC gene and *E. coli* ST combination compared to the cross-sectional study [*bla*_CTX-M-15_ in ST131 (*n* = 3), *bla*_CTX-M-15_ in ST69 (*n* = 1), *bla*_CTX-M-55_ in ST69 (*n* = 1), *bla*_CMY-2 like_ in ST10 (*n* = 1)] (Table 3). In five out of six persons the ESBL gene was located on the same plasmid [IncB/O/K/Z (*n* = 3), IncF (*n* = 1), untypeable plasmid (*n* = 1)], in the sixth person the gene was located on the chromosome. The *E. coli* core genome of these six persons was highly homologous to the genome that was found 5 years earlier, with the number of SNP differences ranging from 46 to 186, as is shown in Fig. 2.

## DISCUSSION

Although meat is presumably the most important food-related source for transmission of ESBL-E/K, our previous research found that persons who consume meat at least three times per week did not have a higher risk of ESBL-E/K carriage compared to persons on a vegetarian diet (22). Since vegetables, fruits, and herbs are known to be contaminated with ESBL-E occasionally, we hypothesized that a high share of consumption of these products could potentially result in substantial exposure of consumers and subsequent foodborne dissemination, especially if these products are consumed raw. The present population study, however, could not pinpoint any individual type of raw or undercooked vegetables, fruits, or fresh herbs as a risk factor for the carriage of ESBL-E/K in the gut of healthy individuals.

Consumption of organic fruits and vegetables was associated with an increased odds of ESBL-E/K in univariable analyses. This is consistent with the findings of Reuland et al. who found that organic vegetables sold in the Netherlands were more often contaminated with ESBL-E than conventionally produced vegetables (20). Several studies from all over the world have investigated the presence of ESBL-E on a wide variety of fruits and vegetables, mostly including vegetables that are typically eaten raw, such as lettuce, tomatoes, cucumber, carrots, spring onions, and several types of herbs (10–16, 26–29). Although the total number of samples included in these studies might be large, the number of samples per product is often low. Since the prevalence of ESBL-E can vary and depends on the country of production, a ranking of the prevalence in fruit and vegetable types is lacking. We observed the highest odds of ESBL-E/K carriage for the consumption of bell pepper, celery, and several types of berries (*P* value < 0.20 in univariable analysis). No literature exists on the presence of ESBL-E on bell peppers sold in the Netherlands. However, a German study hinted that fruit vegetables, which include tomato, pepper, zucchini, and cucumber, often have higher rates of AMR than other vegetables, possibly related to secondary contamination due to touching of the vegetables by consumers (30). ESBL-E have sporadically been found on celery in the Netherlands. Van Hoek et al. found third-generation cephalosporin-resistant Enterobacterales in 9 of 192 blanched celery items tested (4.7%) (18), whereas no ESBL-E was found on celery in two other studies (20, 21). Literature on the prevalence of ESBL-E in fruits in the Netherlands is lacking. A limited number of soft fruits has been tested in other European countries, no ESBL *E. coli* was found in a UK study investigating 80 samples of raspberry, strawberry, and blueberry (27), and a Finnish study found no ESBL-E/K in a selection of 16 raspberry, strawberry, blueberry,and blackberry samples (13). In Ecuador, ESBL *E. coli* was found in strawberries (10/31) and blackberries (4/31), which was probably caused by contamination via wastewater (15).

Interestingly, ESBL-E/K prevalence was similar to the prevalence in the same population measured about 5 years earlier (7.0% and 7.6%, compared to 7.5% in 2015–2017). At the time of the sample collection in 2021 strict containment measures were in place for over a year due to the COVID-19 pandemic, which included increased physical distancing, use of face masks in public spaces, and travel restrictions. We hypothesized that this would decrease the ESBL-E/K carriage rate in the general population, since human contact is regarded the main route of transmission, and travel to countries with a high risk of ESBL-E acquisition has been an established risk factor (5, 31, 32). Indeed, the percentage of participants traveling to Africa, Asia, and Latin America in the preceding 6 months decreased from 13.0% in 2015–2017 to 1.2% in 2021. Furthermore, the frequency of antibiotic use in the preceding 3 months increased slightly from 5.5% in 2015–2017 to 6.0% and 7.5% in summer and fall of 2021, respectively. Although we lack a measurement at the start of the COVID-19 pandemic, our results are in line with an editorial by Monnet et al., in which it was predicted that in Europe determinants resulting in an increase and a decrease in AMR due to the pandemic might be balanced (33). A recent global review and meta-analyses found a non-statistically significant increase for resistant Gram-negative bacteria since the start of the pandemic, although this was based on literature of hospitalized patients and mostly focused on healthcare-acquired resistance (34). In contrast, in Ecuador, a middle-income country that implemented strict policies to reduce COVID-19 transmission, the prevalence of third-generation cephalosporin-resistant *E. coli* in children from semi-rural communities decreased from 40% to 23% between 2018 and 2021 (35).

Remarkably, *bla*_DHA-1_ was the second most prevalent ESBL/pAmpC gene in the present study, after *bla*_CTX-M-15_, but only one participant carried *bla*_DHA-1_ in the same *E. coli* type at both sampling moments. In the same participants in 2015–2017, *bla*_DHA-1_ was found just once (data not shown), although there might have been underreporting since this gene was only screened for in isolates with initially negative PCR results (22). The *bla*_DHA-1_ gene was found only sporadically in another Dutch population study in 2014–2015 (3). This might be an indication that this gene has been emerging recently in the Netherlands. Fifteen persons who were ESBL-E/K positive twice with the same ESBL gene and *E. coli* ST carried ST 10, 38, 69, 131, or 349. All except the latter have been described before as STs that are more likely to cause persistent carriage (36). Of the ESBL-E/K-positive participants in the present study, six were carriers of an ESBL-E/K that was genetically highly homologous to the bacteria found in the cross-sectional study approximately 5 years earlier. This long follow-up period is unique, since longitudinal population studies on ESBL-E usually have follow-up periods up to 1 year (37, 38). Although, in theory, this finding could be the result of repeated uptake from the same source, long-term carriage cannot be ruled out.

We developed a digital FFQ focusing on fruit and vegetable consumption to collect information on dietary intake. An FFQ is an easily administered diet assessment method with a low burden for respondents, which can assess past dietary intake. However, a limitation of this method is that FFQs can be prone to recall bias (39). We attempted to avoid this by limiting the recall period to 7 days. The FFQ was administered four times over a period of 3 months to take into account seasonal differences and to overcome sporadic deviations in intake for instance due to holidays. Another limitation to our study is that colonization with ESBL-E/K usually goes unnoticed and thus could have occurred months before sampling, at a time when a person’s consumption pattern was different. However, this is not only a complication to the current research but for any risk factor analysis on ESBL-E/K carriage. Finally, the presence of bacteria on fresh produce can be diminished by washing. However, in order to keep the number of questions within reasonable limits, we did not ask about the washing frequency of individual products. Instead, we added questions on general kitchen hygiene, including hand washing frequency before food preparation, as a proxy for hygiene during the process of food preparation.

It is likely that dissemination of ESBL-E/K to humans through ingestion of food occurs and contributes to the introduction of new strains. However, the transfer of ESBL-E/K from food intake to colonization of the human gut, including dose–response relations, is still poorly understood. Furthermore, studies monitoring contamination of fruits and vegetables with antimicrobial-resistant micro-organisms at the production and processing stage are scarce. In the present study, we showed that the contribution of the consumption of specific raw or undercooked vegetables, fruits, or herbs to ESBL-E/K carriage in humans in the Netherlands is low. The consumption pattern of small quantities of many different types of fresh produce and the relatively low frequency of ESBL-E contamination of these products, as well as food not being the main route of transmission, all potentially contribute to the lack of effect that was found. Nevertheless, it is recommended to thoroughly wash fresh products especially when they are eaten raw, and to prevent cross-contamination from other products or the hands of the person preparing the food. In addition, the ESBL-E/K prevalence was similar to 5 years earlier, despite COVID-19 containment measures and we demonstrated the presence of genetically highly homologous ESBL-E/K after 5 years in the same individuals, indicative for long-term carriage.

## Data Availability

Sequencing data have been deposited to the ENA (European Nucleotide Archive) under accession number PRJEB63082.

## SUPPLEMENTAL MATERIAL

Supplemental figures and tables spectrum.02849-23-s0001.pdf Instruction leaflet, Tables S1 to S4, and Fig. S1 to S4.

ASM does not own the copyrights to Supplemental Material that may be linked to, or accessed through, an article. The authors have granted ASM a non-exclusive, world-wide license to publish the Supplemental Material files. Please contact the corresponding author directly for reuse.
