# Supplementary material for: Human carriage of ESBL/pAmpC-producing Escherichia coli and Klebsiella pneumoniae in relation to the consumption of raw or undercooked vegetables, fruits, and fresh herbs
Source: Microbiol Spectr. 2024 Jan 11;12(2):e02849-23. doi: 10.1128/spectrum.02849-23 (PMC10845978; doi:10.1128/spectrum.02849-23)
Supplement: Supplemental figures and tables — Instruction leaflet, Tables S1 to S4, and Fig. S1 to S4. [file spectrum.02849-23-s0001.pdf]

Instruction procotol - Fecal sample

Supplies

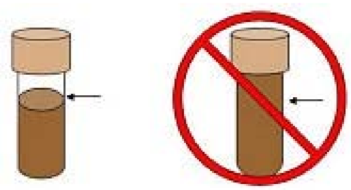

- Sampling tube with spoon
- Absorption sheet
- Transport blister
- Safety bag
- Shipping envelope
- Collection bag
- Disposable gloves

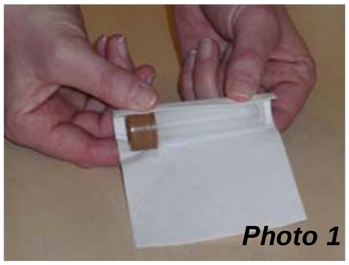

Photo 1

Time of collection

Collect the sample preferably on Monday, Tuesday or Wednesday. Please complete the online questionnaire first.

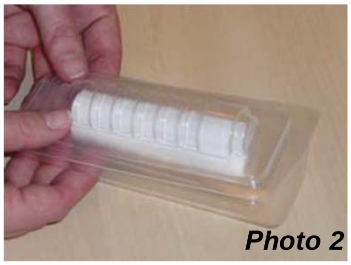

Photo 2

How to collect the fecal sample

1. Write the collection date on the sampling tube.
2. If needed, place the collection bag on your toilet seat.
3. Put on the disposable gloves.
4. Scoop small amount of feces in the sampling tubes using the spoon on the inside of the cap. Fill the tube halfway, not fuller (see image above).
5. Tighten the cap properly to prevent the contents from leaking out, and clean the outside of the tube (with alcohol or soap).

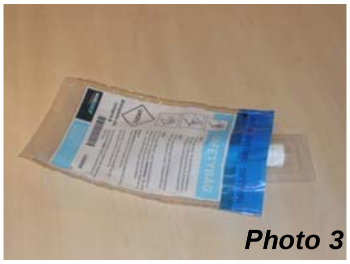

Photo 3

How to pack the fecal sample

6. With clean hands, wrap the tube in the absorption sheet, cover the cap as wel (*photo 1*).
7. Place the tube in the transport blister (*photo 2*) and close properly.
8. Put the blister in the safety bag (*photo 3*) and close it by removing the white closing strip. Make sure to enclose as little air as possible in the safety bag.
9. Place the safety bag in the blue plastic shipping envelope (*photo 4*).
10. Store the package at room temperature until sending.
11. Post the envelope the same day in an orange PostNL mailbox. A stamp is not required.

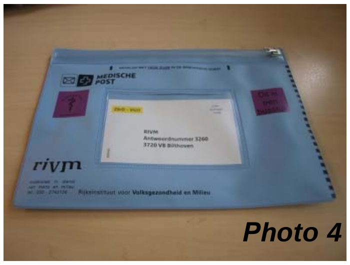

Photo 4

We thank you kindly for your cooperation!

Supplementary table S1. General characteristics based on Questionnaire 1

	N	%
Total	537	100.0
**General**		
Month of participation		
June	39	7.3
July	311	57.9
August	168	31.3
September	19	3.5
Gender		
Male	132	24.6
Female	405	75.4
Age *(median; min-max*)	56	24-88
Urbanization level[1]		
Very high	151	28.1
High/moderate	234	43.6
Low/very low	152	28.3
Country of birth		
Other	21	3.9
Netherlands	516	96.1
Country of birth of participants and both parents		
UNK	1	0.2
Other	60	11.2
Netherlands	476	88.6
Has children (<4 years) who go to day-care	31	5.8
Level of education		
Low/middle	161	30.0
High	376	70.0
Works with children <4 years of age	24	4.5
Works in healthcare	99	18.4
Average number of days per week working on location, with colleagues, costumers, clients or patients, in the last 4 weeks		
0 days	253	47.1
1-2 days	120	22.4
3 or more days	164	30.5
**Diet and hygiene**		
Diet		
Vegetarian	310	57.7

Non-vegetarian	102	19.0
Pescatarian	125	23.3
In what types of shops do you buy fruits and vegetables		
Greengrocer	92	17.13
(Organic) supermarket or delivery service	511	95.16
Asian food store	32	5.96
Deli	8	1.49
(Organic) market	122	22.72
Farm shop	70	13.04
Own (or shared) vegetable garden	82	15.27
How often do you buy organic fruits and vegetables		
I don't know	8	1.49
Always/usually	77	14.34
Regularly/sometimes	279	51.96
Rarely/never	173	32.22
Dairy products are consumed in the household	483	89.94
In what types of shops do you buy dairy products		
Cheese shop	88	16.39
(Organic) supermarket or delivery service	452	84.17
Asian food store	2	0.37
Deli	10	1.86
(Organic) market	79	14.71
Farm shop	40	7.45
How often do you buy organic dairy		
I don't know	58	10.8
Always/usually	132	24.58
Regularly/sometimes	197	36.69
Rarely/never	150	27.93
Fish products are consumed in the household	272	50.65
In what types of shops do you buy fish products		
Fishmonger	88	16.39
(Organic) supermarket or delivery service	203	37.8
Asian food store	3	0.56
Deli	272	50.65
(Organic) market	69	12.85
Local products or self-caught	1	0.19
Meat products are consumed in the household	226	42.09
In what types of shops do you buy meat products		
(Organic) butcher	60	11.17
(Organic) supermarket or delivery service	194	36.13
Asian food store	8	1.49
Deli	1	0.19
(Organic) market	6	1.12
Farm shop	20	3.72
How often do you buy organic meat		

I don't know	324	60.34
Always/usually	55	10.24
Regularly/sometimes	65	12.1
Rarely/never	93	17.32
On average, how many days per week do you eat meat		
0	435	81.0
1-2	35	6.5
3-5	38	7.1
6-7	29	5.4
Has barbecued in the last 4 weeks	40	7.5
Hand washing frequency after toilet use		
Always/usually	397	73.9
Regularly/sometimes	123	22.9
Rarely/never	17	3.2
Hand washing frequency after being outdoors		
Always/usually	242	45.1
Regularly/sometimes	230	42.8
Rarely/never	65	12.1
Hand washing frequency before food preparation		
Always/usually	322	60.0
Regularly/sometimes	179	33.3
Rarely/never	36	6.7
Uses dishcloth for more than 1 day	274	51.0
**Health and medication use**		
Body Mass Index		
UNK	4	0.7
<18.5	17	3.2
18.5-25	362	67.4
25-30	115	21.4
≥30	39	7.3
Hospitalized in last 6 months	21	3.9
Family member hospitalized in last 6 months	13	2.4
Proton pump inhibitor or H2 blocker use in last 6 months	55	10.2
Antibiotic use		
Last 6 months	44	8.2
Last 3 months	32	6.0
Stomach and/or bowel disease		
Gastric mucosa irritation	10	1.9
Acid reflux	61	11.4

Irritable bowel syndrome	41	7.6
Crohn's disease	5	0.9
Stomach and/or bowel complaints in last 4 weeks	89	16.6
Tested positive for COVID-19 in last 4 weeks	2	0.4
**Leisure activities**		
Travel in last 6 months		
No travel, travel to Western/Northern Europe, North America, Australia or New Zeeland	483	94.9
Travel to Southern/Eastern Europe	20	3.9
Travel to Africa, Asia or Latin America	6	1.2
Swimming in fresh water in last 6 months	82	16.1
Swimming in salt water in last 6 months	52	10.2
Swimming in fresh and salt water in last 6 months	20	3.9
Used animal manure in last 6 months	136	25.3
**Contact with animals**		
Owns a pet	265	49.4
Owns a dog	102	19.0
Owns a cat	159	29.6
Lives at a livestock farm/animal company	3	0.6
Has contact with animals at work/school	36	6.7
Works with raw meat	7	1.3
Direct contact with animals in last 4 weeks	394	73.4
Dogs	254	47.3
Cats	242	45.1
Rabbits	40	7.5
Guinee pigs/hamsters	30	5.6
Rats/mice	6	1.1
Birds	17	3.2
Cows	23	4.3
Sheep	21	3.9
Goats	31	5.8
Chicken	36	6.7
Pigs	7	1.3
Horses	52	9.7

1. Very high >= 2500 addresses per km²; high 1500 - 2500 addresses per km²; moderate 1000 - 1500 addresses per km²; low 500 - 1000 addresses per km²; very low < 500 addresses per km²

Supplementary table S2. General characteristics based on Questionnaire 2

	N	%
Total	483	100.0
**General**		
Month of participation		
October	227	47.0
November	219	45.3
December	37	7.7
Average number of days per week working on location, with colleagues, costumers, clients or patients, in the last 4 weeks		
0 days	174	36.0
1-2 days	152	31.5
3 or more days	157	32.5
**Hygiene**		
Hand washing frequency after toilet use		
Always/usually	355	73.5
Regularly/sometimes	116	24.0
Rarely/never	12	2.5
Hand washing frequency after being outdoors		
Always/usually	213	44.1
Regularly/sometimes	212	43.9
Rarely/never	58	12.0
Hand washing frequency before food preparation		
Always/usually	269	55.7
Regularly/sometimes	187	38.7
Rarely/never	27	5.6
**Health and medication use**		
Hospitalized in last 3 months	9	1.9
Family member hospitalized in last 3 months	11	2.3
Proton pump inhibitor or H2 blocker use in last 3 months	49	10.1
Antibiotic use in last 3 months	36	7.5
Stomach and/or bowel complaints in last 4 weeks	106	22.0
Tested positive for COVID-19 in last 4 weeks	5	1.0
**Leisure activities**		
Travel in last 3 months		
No travel, travel to Western/Northern Europe, North America, Australia or New Zeeland	416	86.1
Travel to Southern/Eastern Europe	60	12.4
Travel to Africa, Asia or Latin America	7	1.5
Swimming in fresh water in last 3 months	70	14.5
Swimming in salt water in last 3 months	72	14.9
Swimming in fresh and salt water in last 3 months	27	5.6
Used animal manure in last 3 months	59	12.2
**Contact with animals**		

Direct contact with animals in last 4 weeks	358	74.1
Dogs	236	48.9
Cats	233	48.2
Rabbits	33	6.8
Guinee pigs/hamsters	26	5.4
Rats/mice	7	1.5
Birds	13	2.7
Cows	11	2.3
Sheep	19	3.9
Goats	20	4.1
Chicken	32	6.6
Pigs	7	1.5
Horses	41	8.5

Supplementary table S3. Distribution of ESBL/pAmpC genes and *E.coli/K.pneumoniae* sequence types found in the cross sectional study (2015-2017) and in the present study (2021, Sample 1 and 2)

	**2015-2017 n=40**		**2021 – Sample 1 n=41**		**2021 – sample 2 n=34**	
**ESBL/pAmpC gene**	**n (%)**	**ST (no. isolated)**	**n (%)**	**ST (no. isolated)**	**n (%)**	**ST (no. isolated)**
CTX-M-15	24 (60.0)	10 (1), 38 (2), 69 (3), 131 (5), 196 (1), 349 (2), 398 (1), 517 (1), 540 (1), 636 (1), 648 (2), 1380 (1), 3877 (1), Non-typeable (2)	15 (36.6)	10 (3), 69 (4), 131 (3), 206 (1), 227 (1), 636 (1), 1727 (1), 2325 (1), 4981 (1)	11 (32.4)	10 (2), 38 (2), 69 (2), 131 (3), 382 (1), 409 (1), 504 (1), 517 (1), Non-typeable (1)
DHA-1	1 (2.5)	642 (1)	5 (12.2)	10 (1), 69 (2), 349 (2)	7 (20.6)	10 (1), 38 (2), 156 (1), 349 (2), 708 (1), 1155 (1)
SHV-12	1 (2.5)	226 (1)	4 (9.8)	10 (1), 14 (1), 662 (1), 695 (1), 2308 (1)	1 (2.9)	69 (1)
CTX-M-55	2 (5.0)	69 (1), 457 (1)	2 (4.9)	69 (1), 1429 (1)	4 (11.8)	58 (1), 69 (1), 1193 (1), Non-typeable (1)
CTX-M-27	2 (5.0)	131 (2)	3 (7.3)	69 (1), 131 (1), 1193 (1)	3 (8.8)	23 (1), 69 (1), 131 (1), Non-typeable (1)
CTX-M-14	1 (2.5)	38 (1)	2 (4.9)	10 (1), 38 (1)	2 (5.9)	10 (1), 38 (1)
CMY-2(/22/61)[1]	2 (5.0)	69 (1), 963 (1), 4197 (1)	2 (4.9)	131 (1), 1485 (1)	1 (2.9)	131 (1)
CTX-M-1	4 (10.0)	46 (1), 69 (1), 111 (1), 227 (1)	-		1 (2.9)	69 (1)
CTX-M-32	1 (2.5)	69 (1)	-		-	
CMY-2 like	1 (2.5)	10 (1)	1 (2.4)	10 (1)	1 (2.9)	10 (1)
CMY-7	1 (2.5)	10 (1)	-		-	
CTX-M-1, CTX-M-55	-		2 (4.9)	1429 (2), 1434 (1), 4774 (1)	-	
CTX-M-15, SHV-12	-		1 (2.4)	10 (1)	-	
CTX-M-2	-		1 (2.4)	3672 (1)	-	
CTX-M-8, CTX-M-15 and SHV-32	-		1 (2.4)	58 (1), k1873 (1)	-	
DHA-1 and SHV-187	-		1 (2.4)	k17 (1)	-	

SHV-27	-		1 (2.4)	kNon-typeable (1)	-	
CTX-M-65, DHA-1	-		-		1 (2.9)	106 (1), 349 (1)
SHV-106	-		-		1 (2.9)	k15 (1)
TEM-15	-		-		1 (2.9)	73 (1)

pAmpC, plasmid-mediated AmpC; ST, sequence type
The *K. pneumoniae* sequence types are indicated with a 'k' and underlined, all other sequence types are *E.coli*.
Three isolates were tested per sample, in some participants more than one sequence type or ESBL/pAmpC gene was found.

1. With the primers used in the cross sectional study (2015-2017) no distinction could be made between *bla*CMY-2, *bla*CMY-22 and *bla*CMY-61

Supplementary Table S4. Characteristics of 19 participants that became ESBL-E/K carrier in the second sample or remained positive but with a different ESBL/pAmpC gene or bacterial ST

	**1**	**2**	**3**	**4**	**5**	**6**	**7**	**8**	**9**	**10**	**11**	**12**	**13**	**14**	**15**	**16**	**17**	**18**	**19**
ST sample 1	1485	-	-	-	-	-	14	-	1193	-	-	-	-	-	-	58, k1873	69	-	2026, 2325
ESBL gene sample 1	CMY-2	-	-	-	-	-	SHV-12	-	CTX-M-27	-	-	-	-	-	-	CTX-M-8, CTX-M-15 and SHV-32	DHA-1	-	CTX-M-15
ST sample 2	349	Non-typeable	1193	69	38	58	Non-typeable	73	15	156	23	38, 382, 504	38	349	38	69	10	517	708, 1155
ESBL gene sample 2	DHA-1	CTX-M-55	CTX-M-55	CTX-M-15	DHA-1	CTX-M-55	CTX-M-15	TEM-15	SHV-106	DHA-1	CTX-M-27	CTX-M-15	DHA-1	DHA-1	CTX-M-15	CTX-M-1	DHA-1	CTX-M-15	DHA-1
Gender	1	1	0	1	1	1	0	1	1	0	0	1	1	1	1	0	0	1	1
Age	37	62	73	48	61	35	62	67	54	35	52	24	65	37	41	52	45	38	37
Born in NL	1	1	1	1	1	1	1	1	1	1	1	1	1	1	1	0	1	1	1
Diet (vegetarian: =1; non-vegetarian=2; pescatarian=3)	1	3	2	1	2	2	3	3	1	1	1	1	1	1	3	2	3	1	1
Has contact with animals at work/education	0	0	0	0	1	0	0	0	0	0	0	0	0	0	0	0	0	0	0
Works in healthcare	0	0	0	1	1	1	0	0	0	0	1	0	0	0	1	0	0	0	0
Works with children <4 years of age	0	0	0	0	1	0	0	0	0	0	0	0	0	1	0	0	0	0	0
Works with raw meat	0	0	0	0	0	0	0	0	0	0	0	0	0	0	0	0	0	0	0
Lives at a livestock farm/animal company	0	0	0	0	0	0	0	0	0	0	0	0	0	0	0	0	0	0	0
Owns a pet	1	0	0	1	1	0	0	0	0	0	1	0	0	1	0	1	1	1	0
Hospitalization[1]	0	1	0	UNK	0	0	0	0	0	0	0	0	0	1	0	0	0	0	0

Proton pump inhibitor or H2 blocker use[1]	0	0	0	UNK	0	0	0	0	0	0	1	1	0	0	0	0	0	0	1
Antibiotic use[1]	0	1	0	UNK	0	0	0	0	1	0	0	0	0	1	0	0	0	0	0
Stomach and/or bowel complaints (last 4 weeks)	0	0	0	UNK	0	0	0	0	1	0	0	1	0	1	0	0	0	1	1
Tested positive for COVID-19 (last 4 weeks)	0	0	0	UNK	0	0	0	0	0	0	0	0	0	0	0	0	0	0	0
Travel[1]	1	1	1	UNK	1	1	1	1	1	1	1	2	1	1	1	3	1	1	1
Used animal manure[1]	0	0	0	UNK	0	0	0	0	0	0	0	0	0	0	0	0	1	1	0
Number of days per week working on location, with colleagues, costumers, clients or patients (last 4 weeks)	2	2	1	UNK	3	3	2	2	2	2	3	2	1	1	2	2	2	3	3
Direct contact with animals (last 4 weeks)	1	0	0	UNK	1	0	1	1	1	1	1	1	0	1	1	1	1	1	1
Hand washing frequency after toilet use	1	1	2	UNK	1	2	1	1	1	1	1	1	2	1	1	1	1	1	1
Hand washing frequency after being outdoors	2	1	2	UNK	1	3	2	1	2	2	1	2	1	1	3	2	1	2	1
Hand washing frequency before food preparation	2	1	1	.	1	3	2	1	1	2	1	2	1	2	1	2	2	2	1

pAmpC, plasmid-mediated AmpC; ST, sequence type; UNK, unknown.

1. In the last 3 months (period between the first and second faecal sample)

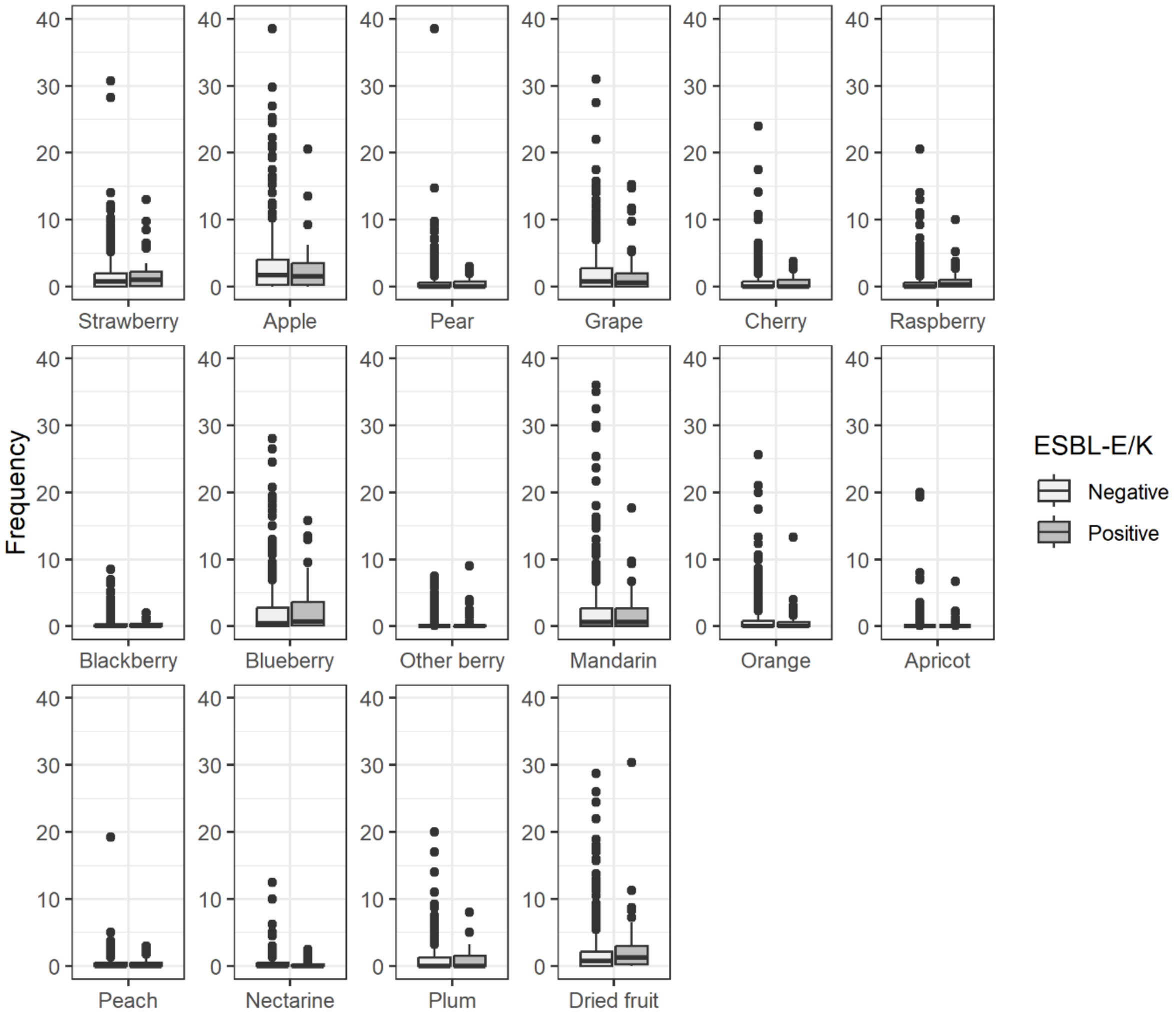

Supplementary figure S1. Box plots of the average weekly consumption of raw fruits (number of hands or pieces), for ESBL-E/K negative (n=436/489) and positive participants (n=53/489). Two outliers are removed (orange (1), apple (1)) with average weekly consumption of >40 pieces.

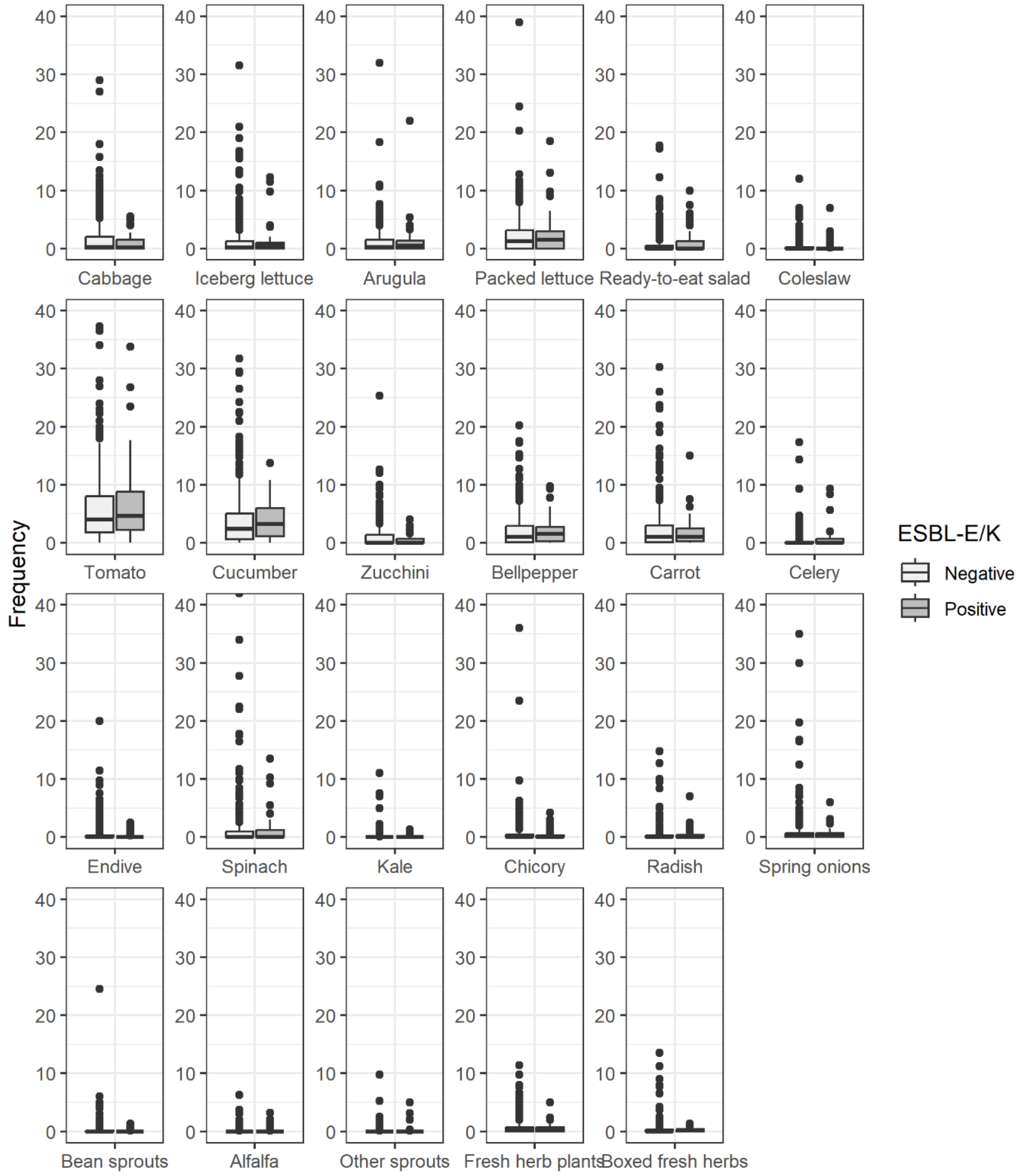

Supplementary figure S2. Box plots of the average weekly consumption of raw or undercooked vegetables and herbs (number of serving spoons), for ESBL-E/K negative (n=436/489) and positive participants (n=53/489). Two outliers are removed (iceberg lettuce (1), spinach (1)) with average weekly consumption of >40 serving spoons.

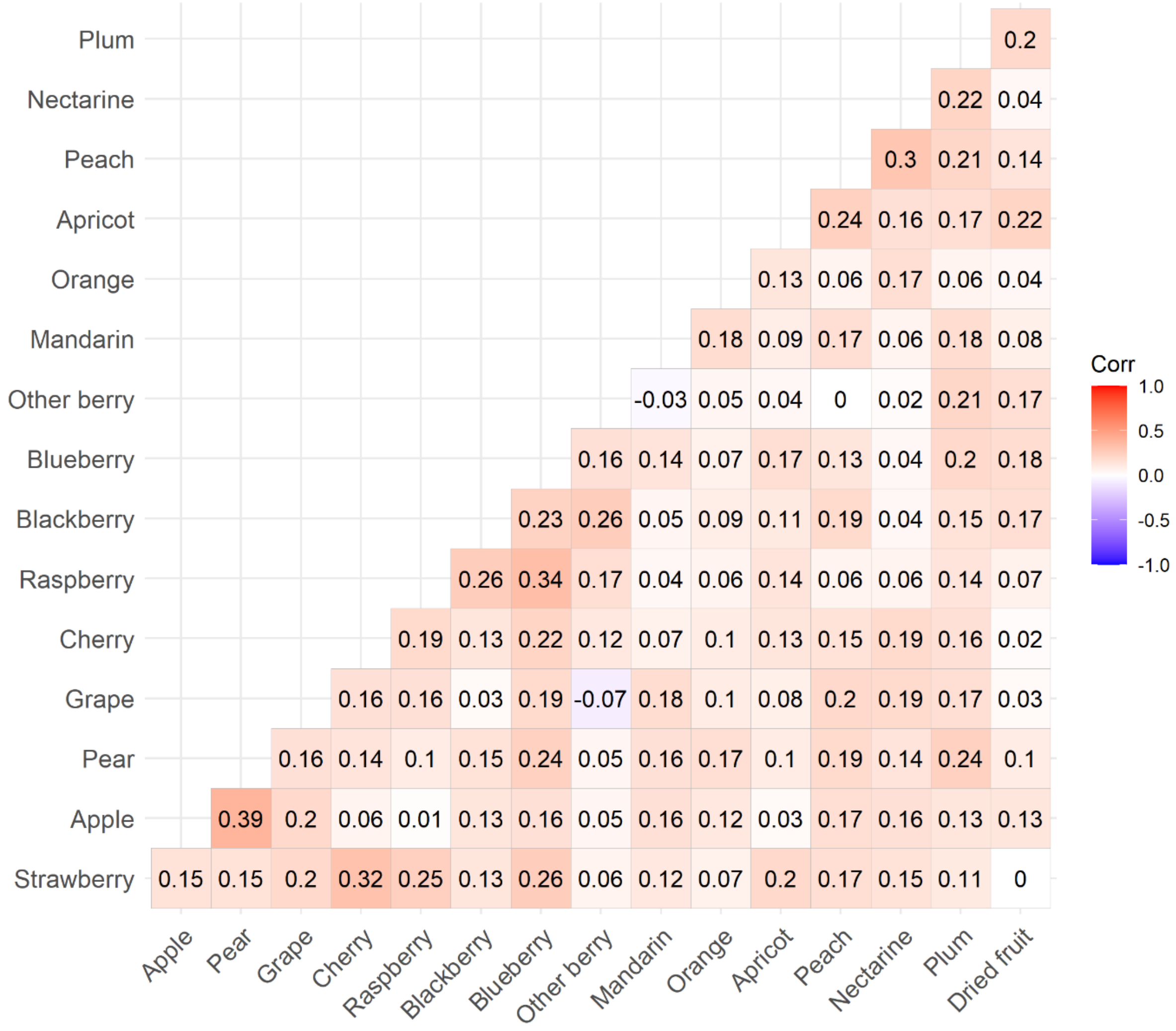

Supplementary figure S3. Fruits correlation matrix (Spearman)

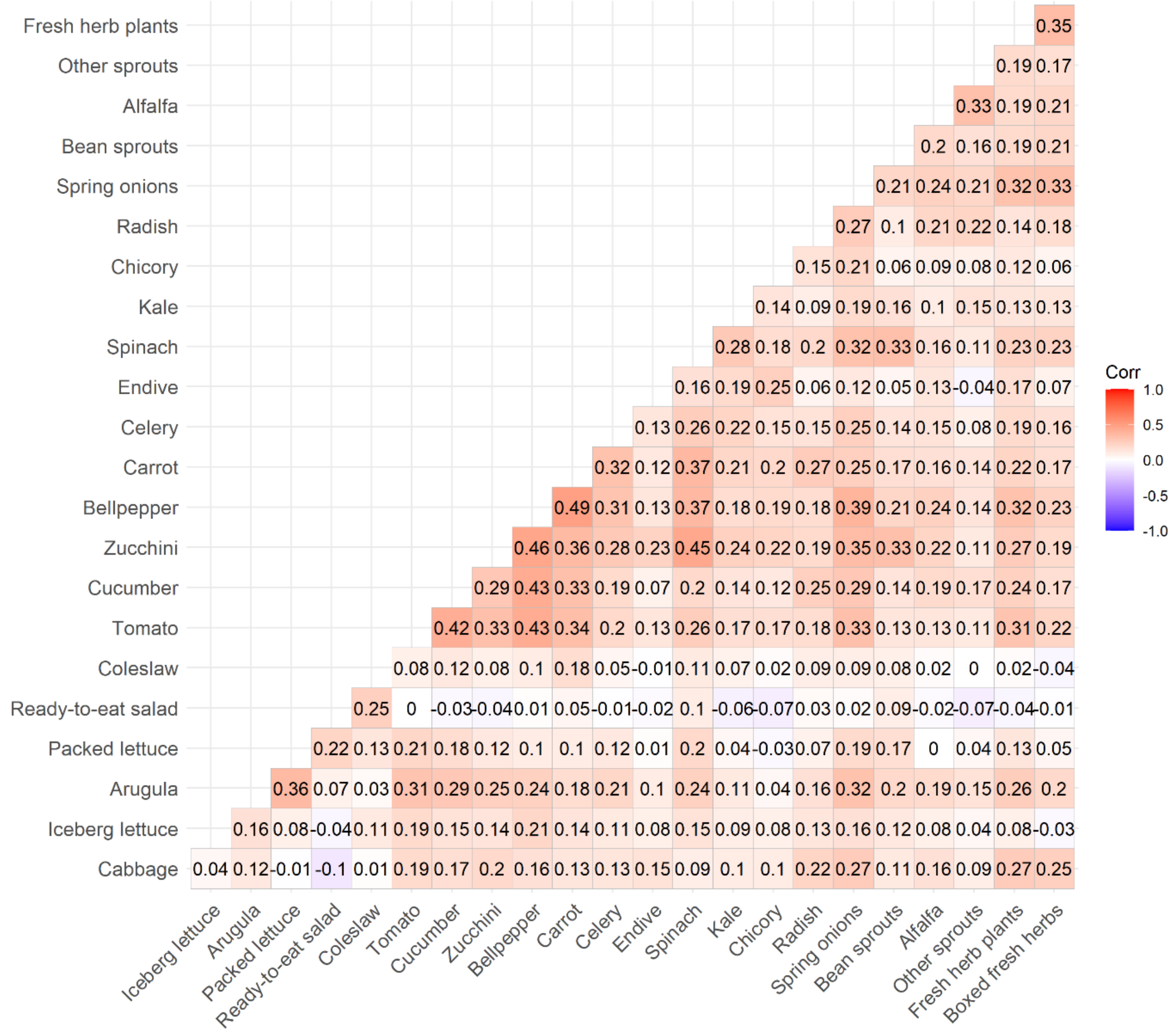

Supplementary figure S4. Vegetables correlation matrix (Spearman)
